# Developing a Systematic Training Programme in Women’s Mental Health

**DOI:** 10.1192/j.eurpsy.2022.171

**Published:** 2022-09-01

**Authors:** L. Mcdonald

**Affiliations:** Royal College of Psychiatrists, Professionalk Standards, London, United Kingdom

**Keywords:** Perinatal psychiatry, post-membership training, consultants, senior trainees

## Abstract

This presentataion outlines the development of a post-membership masterclass programme in Perinatal Psychiatry, funded by Health Education England and delivered through the Royal College of Psychiatrists. The masterclass programme renges from 5-15 days and there are separate programmes for consultants, SAS doctors and senior trainees in psychiatry. The course is delivered by experts in the area and contains a mix of didactic teaching and small group work. The programme was developed to meet the workforce needs of rapidly expanding perinatal mental services throughout England. The programme also helps facilitate the needs of perinatal psychiatrists from Ireland and from the devolved nations of the UK (Scotland, Wales and Northern Ireland).

**Disclosure:**

No significant relationships.

